# QbTest for Monitoring Medication Treatment Response in ADHD: A Systematic Review

**DOI:** 10.2174/0117450179276630231030093814

**Published:** 2023-11-01

**Authors:** Urban Gustafsson, Mikkel Hansen

**Affiliations:** 1 Qbtech AB, Medical Department, Cardellgatan 1, 11436 Stockholm, Sweden

**Keywords:** ADHD, QbTest, Medication, Monitoring, Treatment, Drugs, Psychopharmacology

## Abstract

**Introduction:**

Attention deficit hyperactivity disorder (ADHD) is considered one of the most common neurodevelopmental disorders in childhood and adolescence. Pharmacological treatment plays an important part in the therapy of the disorder and verifying the effectiveness of ADHD medication is essential throughout the course of treatment. QbTest is a computerized test, for which intended use is to provide healthcare professionals with objective measurements of hyperactivity, impulsivity, and inattention to aid in the clinical assessment of ADHD and the evaluation of treatment interventions.

**Methods:**

A systematic review of relevant articles was conducted for which QbTest was used for monitoring medication treatment response in ADHD. Literature published between 2004 and 2023 was appraised.

**Results:**

A total of 15 studies were included in the review. Thirteen articles involved subjects diagnosed with ADHD and two studies that were related to the disorder, which evaluated QbTest in medication treatment response. Changes in QbTest data such as Q-scores, effect size, or improvement/deterioration of QbTest variables were evaluated. A clinically relevant decrease in QbTest Q-scores was found in the majority of the studies when treated with any type of ADHD medication in therapeutic doses, both in comparison to placebo and when compared from baseline to endpoint treatment.

**Conclusion:**

QbTest can distinguish pharmacological treatment effects within hours of pharmacological titration and can be used for monitoring of long-term treatment of ADHD. A need for optimization and individualization of medication treatment response could be addressed with access to objective measures in ADHD management.

## 1 INTRODUCTION

Attention deficit hyperactivity disorder (ADHD) is a prevalent and persistent disorder that emerges early in childhood, with a current prevalence rate of approximately 5% [1, 2]. ADHD is believed to have its onset in early childhood, although it is typically not diagnosed before the school-age years and is considered one of the most common mental health conditions in childhood and adolescence [1, 3]. ADHD is characterized by three core symptom domains: inattention, hyperactivity, and impulsivity, for which all domains are important to distinguish in any patient for a diagnosis and management of ADHD.

Insufficiently treated ADHD can have many consequences such as job instability, drug- and alcohol abuse, social functioning, relations, family functioning, increased healthcare costs [4, 5] as well as increased mortality rate [6]. For certain comorbid conditions, undiagnosed and untreated ADHD can lead to suboptimal outcomes of higher costs than if ADHD and comorbid conditions were treated separately [7, 8]. Medication compliance is a common problem in ADHD treatment which may hamper the course of pharmacological treatment [9]. Stimulant treatment for ADHD has increased in the last two decades [10], and approximately 50% of the children (in the US) have been reported being treated with pharmacological medication [10, 11]. Thus, the evaluation of pharmaceutical treatment is important in ADHD [12].

The QbTest consists of a high-resolution motion tracking system combined with a computerized Go/No-Go paradigm for children. The Go/No-Go paradigm is based on a task in which participants must press a handheld responder button each time a circle appears on-screen but withhold the response when a cross appears in front of the circle. For adolescents and adults, a one-back task that involves four types of stimuli the target stimulus is defined as the stimulus that is identical in shape and color to the one preceding it. Physical activity is measured during the test performance via an infrared camera that tracks the path of a reflection attached to the center of the participant’s forehead. The elements of the test are visually displayed in a report that provides information on each of the three symptom domains of ADHD (QbActivity, QbInattention, QbImpulsivity). Summary scores for each individual are based on deviations from a normative dataset based on age group and gender [13-18]. The QbTest thus separately assesses all three core symptoms of ADHD on a behavioral level [18-21]. The results from the test are presented as raw scores as well as percentiles and Q-scores that are calculated using an age and gender-adjusted norm group. The percentile expresses the probability of a normative person scoring lower than the test person. Therefore, a test result that ends up in the 93^rd^ percentile shows that 93% of the normative group scores lower than that test person. This corresponds to a standard deviation of 1.5 (Q-Score) [22] which could be described as atypical. The Q-score of 1.1 is equivalent to the 86^th^ percentile, which means that 86% of the normative group will score lower than the person who has obtained these results on the test. Thus, a Q-score ranging from 1.1 to 1.4 is reflected as slightly abnormal. A total Q-score (mean of all cardinal parameters of QbTest; QbActivity, QbInattention and QbImpulsivity) reduction of -0.5 (half standard deviation) is considered a clinically significant improvement [23].

The aim of this systematic review was to examine the literature that evaluates QbTest’s ability to detect changes in test performance as a measure of treatment response when monitoring medication effects in ADHD.

## 2 METHODS

We searched the following electronic databases: PubMed, Embase and Cochrane Library for relevant studies published from 2004 through July 2023 (QbTest was approved by FDA 2004). The selection procedure of articles followed the guidelines of the PRISMA method [24]. The searched terms were chosen using cross-matched keywords combinations: “qbtest”, “cb-cpt”, “attention deficit disorder with hyperacti-vity”, “adhd”, “quantitative behavior test”, “quantitative behav-ioral test”, “treatment”, “monitoring”, “pharmacological”, “drug”, “drugs”, “atomoxetine”, “guanfacine”, “viloxazine”, “methylphenidate”, “dexmethylphenidate”, “amphetamine”, “amfetamine” “lisdexamphetamine”, “lisdexamfetamine”, and “psychopharmacology”.

The inclusion criteria were a) Clinical studies that evaluated the monitoring of medical treatment intervention using QbTest in assessing treatment response b) Subjects of both genders aged ≥ 6 years c) Reference standards for ADHD diagnosis that should be based on a clinical diagnosis according to the DSM or ICD criteria or equivalent standard, e) Selections on peer-reviewed journal articles in the English language with no restriction on clinical study geographical location. The exclusion criteria were a) Review articles and/or meta-analysis, b) Conference abstracts and c) Studies based on solicited diagnosis.

## 3 RESULTS

**Fig. (1):**
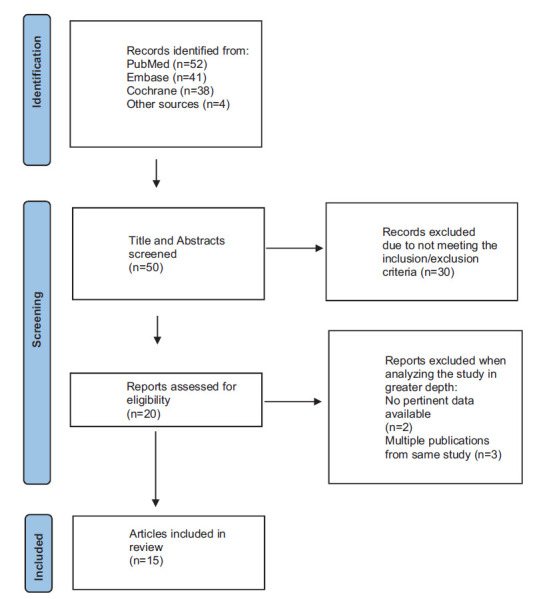
Flow chart of the search process.

A total of 50 unique articles were identified from the search, 30 studies did not meet eligibility criteria and 20 articles were assessed for eligibility criteria (Fig. **1**). Three articles were duplicate publications, and two studies were inconclusive (no pertinent data), leaving 15 articles to be included in this review. Thirteen articles included subjects diagnosed with ADHD and two studies related to hyperkinetic disorder and Autism Spectrum Disorder evaluated QbTest in medication treatment.

A description of the included studies is provided, in alphabetical order, in Table **1**. Fig. (**2**) shows QbTest performance changes across all 15 studies in three groups based on QbTest derived performance measures. In each group the studies are sorted based on follow up time and performance is either change from baseline or compared to placebo. Four studies showed statistically significant reductions in total Q-score [23, 25-27]. In three studies a statistically significant improvement could be detected from the effect size estimates [28-30]. In six studies a statistically significant enhancement was found in QbTest variables or cardinal domains after medication treatment [31-36]. In one study the total Q-score was reduced [37] (no statistical significance calculation could be made as the QbTotal data was calculated manually from this article), and in one study the total Q-score did not change [38].

## 4 DISCUSSION

### 4.1 Evaluation of the Study

The number of patients ranged from 30-364 in the included studies, which could be considered a satisfactory sample size for diagnostic evaluation [39]. All studies were performed in Europe, except one study which was completed in the US. Methylphenidate was the most common medication used in the treatment of ADHD in our review (12/15 studies). In Europe, methylphenidate either as short- or long-acting preparation is the first-line medication for ADHD across the lifespan [40]. Second-line medications are lisdexamfetamine, atomoxetine, and guanfacine [40], and these drugs were occasionally used in our findings as well. Age distribution in the studies ranged from 6 to 61 years. The duration of the treatment and QbTest assessments were between 1 day up to 4 years in the assessed publications, of which 3 studies had a duration ≥12 months. Two serious adverse events were reported (muscular spasm, and increased heart rate and breathing) in one study with cannabinoid treatment [38], and one study reported a serious adverse event (unspecified with unknown cause) with methylphenidate treatment [28], but none were related to the device. Otherwise, any medical treatment was regarded as generally well-tolerated or no adverse events were presented in the result section of the publications and no adverse events were found related to the QbTest medical device.

### 4.2 Effect of Mixed or Alternative Treatments Evaluated by QbTest

The long-term effectiveness of different ADHD medications (methylphenidate, lisdexamfetamine, guanfacine, or atomoxetine) in children for a duration of one year was investigated by Cedergren *et al*. [25]. No information on medication type and dose levels was published. QbTest results demonstrated a reduction in symptoms on all cardinal parameters between baseline and after 1 month (p<0.01) as well as after 12 months (p<0.01) of treatment with stimulants. The reductions in total Q-score were -0.56 and -0.83 after 1 month and 12 months, respectively. A similar improvement of symptoms was observed with the ADHD-RS. There was a weak but significant correlation between the total change scores of QbTest and ADHD-RS from baseline to 1 month (r=0.28, p<0.05) but not after 12 months of remedy [25]. Martin-Key *et al*. evaluated the clinical utility of the combined use of objective (QbTest) and subjective symptom measures of ADHD before and after treatment in adults [26]. Methylphenidate, amphetamine, lisdexamphetamine, dextroamphetamine-amphetamine or atomoxetine were given for 6 months, and dose titration was completed 2-5 weeks after baseline. The mean total Q-score decreased by -1.08 after 2-5 weeks of treatment (p<0.001), and decreased by -1.46 after 6 months of treatment compared to baseline (p<0.001), for which 86% of the patients showed a ≥0.5 reduction in total Q-score. Improvement could also be seen in Quality of Life after 6 months (p<0.001), for which a correlation between QbTest total Q-score and Quality of Life total score (AAQoL) at 6 months after treatment initiation (r=0.41, p<0.001) [26].

A naturalistic study by Nylander *et al*. aimed to map QbTest performance in a group of patients already diagnosed with ADHD investigated if those patients who missed their medication performed worse than those who took their prescribed medication at baseline and at a follow-up four years later [34]. Most patients were prescribed more than one add-on drug to their stimulant. Patients who tested twice on QbTest performed significantly better on the follow-up test 4 years later compared with baseline test on all three cardinals (p<0.05) [34].

In a pilot randomized placebo-controlled study (Cooper *et al*.), the effect of cannabis was investigated in adult patients diagnosed with ADHD for a duration of 6 weeks [38]. An estimated difference in total Q-score of -0.17 between active and placebo was found but did not reach statistical significance [38].

### 4.3 Medication Treatment Approach

Treatment of ADHD often requires a multimodal approach, such as behavioral therapies or pharmacological treatments. Pharmacological treatment of ADHD has been found to be positively associated with improved achievement in academic elementary school children and improved health-related quality of life in children and adolescents [41]. Treatments with available drugs include stimulants (such as methylphenidate, and amphetamines) and non-stimulants (such as atomoxetine, and guanfacine) that may well give satisfactory management of the disease on a short-term basis but depending on the tolerability of the drugs, long-term use can be challenging [9, 42]. Medication adherence could be a problem in individuals with ADHD, and when any pharmacological treatment is discontinued for whatever reason, it could be detrimental to the quality of life, especially in the younger population [43]. An increased risk of substance abuse may also limit long-term pharmacological treatment and limited tolerability should always guide the pharmacological management of ADHD. On the other hand, early and optimal treatment of ADHD has the potential to change the trajectory of psychiatric morbidity later in life and to substantially improve functional outcomes across the spectrum of psychiatric comorbidities [8]. Hall et al. also emphasized that the clinical utility of objective testing in aiding medication management in the clinical practice of ADHD is underinvestigated [13].

### 4.4 Strengths and Limitations

Each parameter included in the cardinal domain from the QbTest is weighted differently depending on its correlation (factor loading) to that cardinal parameter. The cardinal domains QbActivity, QbInattention and QbImpulsivity, were overall derived from the 10-13 variables from the data output of the QbTest from the publications. Thus, one limitation of the compilation of the Q-score data in this review is that in some instances, the calculations of the cardinal scores were based on different specified variables (within the domain) between studies, which may have influenced not only the presented cardinal domain score but also the total Q-score results as well. A further constraint is that the presentation of the total Q-score, when available and presented in the publication, was in some cases based on the mean or the median of the three cardinal domains, and in one case based on the mean of two of the cardinal parameters. Also, the baseline Q-scores were different between the studies (ranging from >1 to > 2). These considerations in part or together may have influenced the medication treatment responses found as measured by the QbTest. Furthermore, the heterogeneity between the studies as well as the presented data in the publications made it difficult to pool the output of the QbTest data. This review was not a meta-analysis, and its findings could be misled by publication bias that was not assessed. Integrating data from studies using meta-analytical methods may refine the statistical precision better than the description of an individual study [44].

## CONCLUSION

The purpose of this article was to conduct a review of publications evaluating QbTest, an objective measure, when used as an aid in monitoring medication treatment response in ADHD subjects. A decrease in QbTest Q-scores in the clinical magnitude range was found in the majority of the studies when treated with any type of ADHD medication in therapeutic doses, both in comparison to placebo and compared from baseline to endpoint treatment. This pattern was seen both in short-term (over course of a day) and in long-term (≥ one year) studies. Based on these findings our conclusion is that QbTest can distinguish medication treatment effects within hours of dosing and can also be used as an aid in the monitoring of long-term treatment of ADHD. A benefit could lie in the access to unbiased data and the interpretation of objective measures in the context of subjective information. This could open for a much more standardized and faster treatment titration. Future studies are warranted to evaluate QbTest and monitor treatment interventions in the ADHD clinical workflow.

## Figures and Tables

**Fig. (2):**
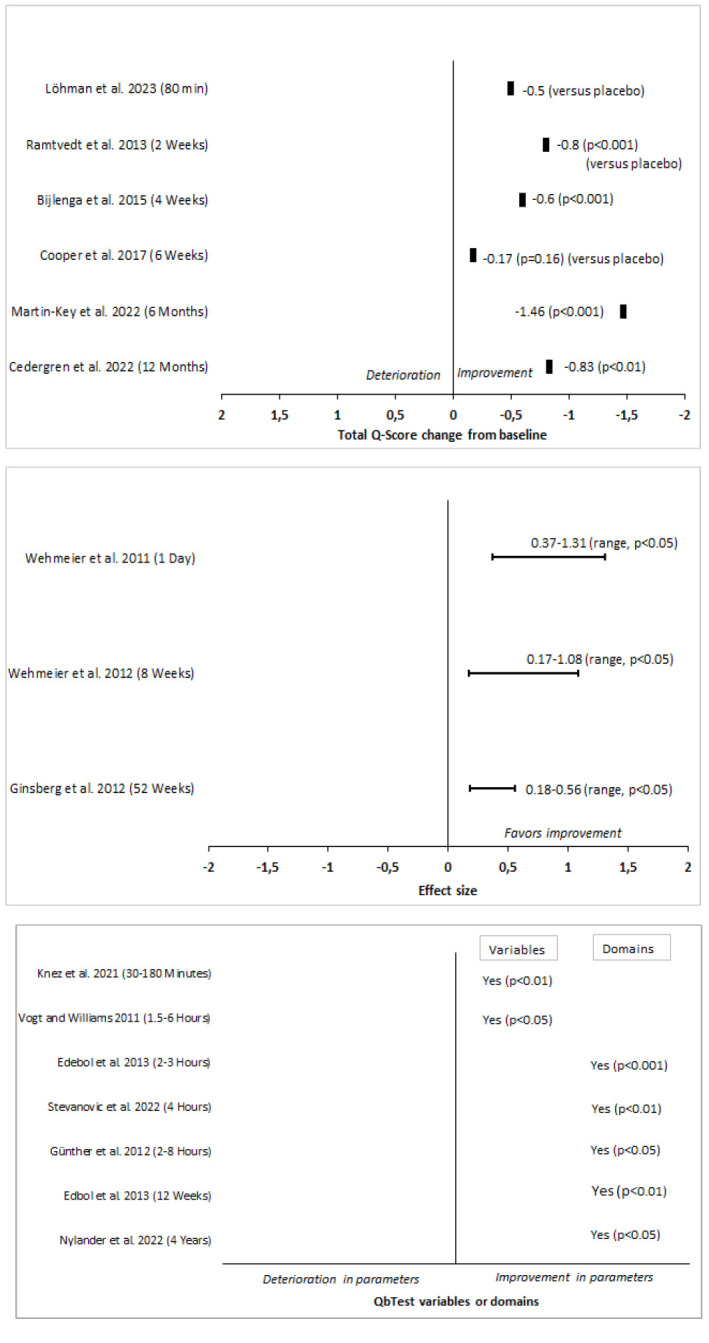
QbTest total Q-score change from baseline (upper panel), effects size ranges (middle panel) and improvement/deterioration in QbTest variables (time active, distance, area, microevents, reaction time, reaction time variation, normalized variation, omission error, commission error, error rate/motion simplicity) or domains (QbActivity, QInattention and QbImpulsivity) (lower panel) from QbTest juxtaposed to the duration of medication treatment. Data obtained from respective author article (Löhman *et al.* n=40, Ramtvedt *et al*. n=36, Bijlenga *et al*. n=82, Cooper *et al*. n=30, Martin-Key *et al*. n=71, Cedergren *et al*. n=78, Wehmeier *et al*. n=125, Ginsberg *et al*. n=30, Knez *et al*. n=364, Vogt & Williams n=44, Edebol *et al*. n=63 and n=10, Stevanovic et al. n=343, Günther *et al*. n=56, Nylander *et al*. n=41).

**Table 1:** An overview of studies using QbTest as a part of monitoring medication treatment response in subjects with ADHD and related disorder.

**Author/Refs.**	**Title of the Article**	**Sample Size**	**Age Years (mean, SD)**	**Medication** **(Dose, Mean, SD)**	**Duration of Treatment**	**Titration Scheme**
Bijlenga (2015) [23]	Objective QbTest and subjective evaluation of stimulant treatment in adult attention deficit-hyperactivity disorder	82	31.3 (10.2)	Methylphenidate (mean dose: 36.3 (16.0) mg) (n=76) Dexamphetamine (mean dose 15.8 (8.5) mg) (n=6)	4 Weeks	A follow-up (after 4 weeks) was made in case the dose was increased: Methylphenidate (mean dose: 49.9 (16.2) mg (n=16) Dexamphetamine (dose: 40.0 mg (n=1)
Cedergren (2022) [25]	Monitoring medication response in ADHD: what can continuous performance tests tell us	78	12.5 (3.6)	ADHD medication (methylphenidate or lisdexamfetamine n=72, guanfacine (n=5) or atomoxetine (n=1))	12 Months	No doses or titration scheme is given in the article.
Cooper (2017) [38]	Cannabinoids in attention-deficit/hyperactivity disorder: A randomized-controlled trial	30	37.9	Spray (100 µL) containing 2.7 mg delta-9-tetrahydrocannabinol (Δ9-THC) and 2.5mg cannabidiol Mean (SD) sprays/day 4.7 (3.3) Placebo	6 Weeks	Two-week titration period;
Edebol (2013) [31]	The Weighed Core Symptom Scale and prediction of ADHD in Adults - Objective measures of remission and response to treatment with methylphenidate	Study I: 63 Study II: 10	35.2 (11.9)	Study I: Methylphenidate: single dose of 13.7 mg (7 mg) Study II: Methylphenidate 18-72 mg	2-3 Hours after intake (Day 1) 12 Weeks	Single dose. Start dose of 18 or 27 (n=10) mg, could go up to 72 mg (n=6) during treatment. Titration 1-4 weeks interval.
Ginsberg (2012) [28]	Long-term functional outcome in adult prison inmates with ADHD receiving OROS-methylphenidate	30	34.4 (10.7)	Methylphenidate (osmotic release oral system) 1.3 mg/kg (36-72 mg)	52 Weeks (5 Weeks (methylpenidate or placebo), this was then followed by an open-label 47 Weeks with methylphenidate)	Start dose 36 mg daily for 4 days, then 54 mg daily for 3 days, then to 72 mg daily for the remaining 4 weeks. During the open-label extension (47 Week), methylphenidate was titrated from 36 mg daily to an optimal dose, with a maximum daily dose of 1.3 mg/kg body weight
Günther (2012) [32]	Modulation of Attention-Deficit/Hyperactivity Disorder symptoms by short- and long-acting methylphenidate over the course of a day	56	10.2-10.9 (1.2-1.9)	Methylphenidate-Immediate Release (MPH-IR) (peak plasma 30 min) (dose: 0.96 (0.15) mg/kg/day) (n=18) Methylphenidate-long acting (MPH-LA) (peak plasma 3-4 h) (dose 0.90 (0.15) mg/kg/day) (n=18) Control (n=20)	2-8 Hours (Day 1)	MPH-LA dose was given in morning; MPH-IR doses were spread over the day; After 7 hours conc. of both formulations was 4 ng/mL and after 12 hour conc. was 2 ng/mL. Treatment started with initial dose of 5 mg, and increased in 5 mg steps until best clinical response obtained
Knez (2021) [33]	The impact of methylphenidate on QbTest performance of children with ADHD: A retrospective clinical study	364	13.6 (3.4)	Methylphenidate: 31.4 (9.5) mg (range 10-72 mg)	30-180 min (Day 1)	Single dose, assessment made in range 30-180 min after intake of methylphenidate
Löhman (2023) [37]	Contrasting expectancy effects with objective measures in adults with untreated ADHD during Qbtest	40	34 (10)	Methylphenidate: 20 mg Placebo	80 min (Day 1)	Single dose, assessment before and after 80 min after intake of pill
Martin-Key (2022) [26]	Investigating the clinical utility of the combined use of objective and subjective measures of ADHD during treatment optimization	71	36.0 (9.7)	Methylphenidate 44.6 (18.6) mg (n=5); Amphetamine 15.2 (3.1) mg (n=13); Lisdexamphetamine 37.8 (16.7) mg (n=38); Dextroamphetamine-amphetamine 30.4 (16.5) mg (n=14); Atomoxetine 100 mg (n=1)	6 Months	Dose titration was made 2-5 weeks after baseline.
Nylander (2022) [34]	The Quantified Behavioural Test Plus (QbTest+) in adult ADHD	67	36 (interquartile 17)	Most patients received metylphenidate and dexamphetamine along with other psychological drugs to mitigate comorbid disorders (medication at baseline n=54, medication at 4 years n=29)	Baseline (Day 1), and follow-up after four years	Baseline and follow-up after four years
Ramtvedt (2013) [27]	Clinical gains from including both dextroamphetamine and methylphenidate in stimulant trials	36	11.4 (1.4)	Methylphenidate: 10 mg Dextroamphetamine: 5 mg Placebo	2 Weeks (Two weeks treatments on each drug randomly in a cross-over design)	Low dose given 1^st^ week, and high dose given 2^nd^ week.
Stevanovic (2022) [35]	ASD with ADHD vs. ASD and ADHD alone: a study of the QbTest performance and single-dose methylphenidate responding in children and adolescents	343	ASD: 14.6 (2.5) ASD/ADHD: 13.0 (3.4) ADHD: 12.6 (3.3)	Methylphenidate: 34.9 (7.3) mg (n=28) 31.4 (13.3) mg (n=95) 30.6 (10.1) mg (n=220)	4 hours (Day 1)	Assessment made 4 hours after administration of methylphenidate
Wehmeier (2011) [29]	Neuropsychological outcomes across the day in children with Attention-Deficit/Hyperactivity Disorder treated with atomoxetine: Results from a placebo-controlled study using a computer-based continuous performance test combined with an infra-red motion-tracking device	125	9.0 (1.9)	Atomoxetine: 1.2 mg/kg/day Placebo	One day (measurements made on morning, noon and evening)	Measurement across the day as such was made on Week 8
Wehmeier (2012) [30]	Does atomoxetine improve executive function, inhibitory control, and hyperactivity	125	9.0 (1.9)	Atomoxetine: 1.2 mg/kg/day. Placebo	8 Weeks	Atomoxetine starting dose was 0.5 mg/kg/day for 1 week, followed by 7 weeks of target dose of 1.2 mg/kg/day.
Vogt and Williams (2011) [36]	Early identification of stimulant treatment responders, partial responders and non-responders using objective measures in children and adolescents with hyperkinetic disorder	44	Children: 10.2 (1.6) Adolescents: 15.8 (1.6)	Methylphenidate-immediate (MPH-IR) release: 0.3 mg/kg Methylphenidate-extended release (MPH-ER): 1.25 mg/kg (mostly adults received this dose)	1.5-6 hours	MPH-IR: assessments made 1.5-2.0 hours post intake; MPH-ER: assessments made 5-6 hours post intake.

## Data Availability

The authors confirm that the data supporting the findings of this review are available within the article.

## SUPPLEMENTARY MATERIAL

PRISMA checklist is available as supplementary material on the publisher’s website along with the published article. Supplementary material is available on the publisher’s website along with the published article.
